# An ovine in vitro model for chondrocyte-based scaffold-assisted cartilage grafts

**DOI:** 10.1186/1749-799X-7-37

**Published:** 2012-11-09

**Authors:** Michaela Endres, Katja Neumann, Bei Zhou, Undine Freymann, David Pretzel, Marcus Stoffel, Raimund W Kinne, Christian Kaps

**Affiliations:** 1 TransTissue Technologies GmbH, Charitéplatz 1, Berlin, 10117, Germany; 2 Tissue Engineering Laboratory, Department of Rheumatology and Clinical Immunology, Charité-Universitätsmedizin Berlin, Charitéplatz 1, Berlin, 10117, Germany; 3 Institute of General Mechanics, RWTH Aachen, Templergraben 64, Aachen, 52062, Germany; 4 Experimental Rheumatology Unit, Department of Orthopedics, University Hospital Jena, Waldkrankenhaus, Rudolf Elle, Klosterlausnitzer Str. 81, Eisenberg, 07607, Germany

**Keywords:** Cartilage grafts, Chondrocytes, In vitro model, Scaffolds, Fibrin, Polyglycolic acid, Tissue engineering, Regenerative medicine, Differentiation, Biomechanics

## Abstract

**Background:**

Scaffold-assisted autologous chondrocyte implantation is an effective clinical procedure for cartilage repair. From the regulatory point of view, the ovine model is one of the suggested large animal models for pre-clinical studies. The aim of our study was to evaluate the in vitro re-differentiation capacity of expanded ovine chondrocytes in biomechanically characterized polyglycolic acid (PGA)/fibrin biomaterials for scaffold-assisted cartilage repair.

**Methods:**

Ovine chondrocytes harvested from adult articular cartilage were expanded in monolayer and re-assembled three-dimensionally in PGA-fibrin scaffolds. De- and re-differentiation of ovine chondrocytes in PGA-fibrin scaffolds was assessed by histological and immuno-histochemical staining as well as by real-time gene expression analysis of typical cartilage marker molecules and the matrix-remodelling enzymes matrix metalloproteinases (MMP) -1, -2 and −13 as well as their inhibitors. PGA scaffolds characteristics including degradation and stiffness were analysed by electron microscopy and biomechanical testing.

**Results:**

Histological, immuno-histochemical and gene expression analysis showed that dedifferentiated chondrocytes re-differentiate in PGA-fibrin scaffolds and form a cartilaginous matrix. Re-differentiation was accompanied by the induction of type II collagen and aggrecan, while MMP expression decreased in prolonged tissue culture. Electron microscopy and biomechanical tests revealed that the non-woven PGA scaffold shows a textile structure with high tensile strength of 3.6 N/mm^2^ and a stiffness of up to 0.44 N/mm^2^, when combined with gel-like fibrin.

**Conclusion:**

These data suggest that PGA-fibrin is suited as a mechanically stable support structure for scaffold-assisted chondrocyte grafts, initiating chondrogenic re-differentiation of expanded chondrocytes.

## Background

Articular cartilage has a limited intrinsic potential for regeneration and repair. Traumatic or focal degenerative defects are often associated with pain, loss of mobility, stiffness and considerable limitations in quality of life and may lead to severe osteoarthritis (OA) and total knee replacement. Since cartilage defects occur frequently, they represent a major and challenging health problem. In knee arthroscopies up to 63% of the patients with knee related symptoms show chondral or deeper osteochondral defects, comprised of approximately 70% focal non-degenerative defects and 30% osteoarthritic defects [1-3]. It is suggested that eleven percent of all knee arthroscopies show cartilage defects suitable for cartilage repair procedures [4].

Clinically applied cartilage repair procedures comprise debridement, bone marrow stimulating techniques like drilling or microfracturing, osteochondral autograft transfer and autologous chondrocyte implantation (ACI) [5-9]. In ACI, a small cartilage biopsy is harvested from the less weight-bearing area of the articular cartilage. After isolation of the chondrocytes by enzymatic digestion of the cartilage extracellular matrix, the cells are grown in vitro in the presence of autologous serum. For chondrocyte implantation, the cartilage defect is debrided down to the subchondral bone and a periosteal flap or a collagen sheet is sutured onto the healthy cartilage. Thus, a self-contained compartment is created that is subsequently filled with the chondrocyte cell suspension by injection. In recent years, a variety of clinical studies documented the clinical efficacy of in vitro expanded chondrocytes for cartilage repair [10-13]. However, there is no significant evidence that the ACI procedure is superior to other cartilage repair techniques in the treatment of full-thickness articular cartilage defects [8,14]. Nevertheless, ACI is regarded as a second line treatment for small and a first line treatment for defects larger than 2 to 4 cm^2^[15].

The inherent technical disadvantages of ACI, such as the need for an intact cartilage rim surrounding the defect, the loss of chondrocytes into the joint space, loosening or ablation of the periosteal flap, and periosteal hypertrophy, may result in re-operations that are reported in up to 25%-36% of the cases [16,17]. Therefore, in recent years, matrix- or scaffold-assisted cartilage tissue engineering grafts were developed that use resorbable, three-dimensional scaffolds and the known regenerative potential of autologous chondrocytes. For hyaluronan-based [18,19], collagen-based [20,21], and resorbable polymer-based [22] autologous chondrocyte grafts, clinical effectiveness for the repair of cartilage defects has been shown for follow-up periods of two to five years.

The regulatory pathways for the approval of such cell-based products, as set by the US Food and Drug Administration, FDA [23] or the European Medicines Agency (EMA) have implications in the development of autologous chondrocyte grafts for orthopedic surgery. The EMA guideline on human cell-based medicinal products summarizes the issues to be addressed during the pre-clinical development of cell-based, hence chondrocyte-based grafts [24]. Pre-clinical studies should address both proof-of-principle and the definition of pharmacological and toxicological effects predictive of the human response, including e.g. quality parameters of the cell-based product, dose-finding, route of administration, application schedule and adverse effects. From the quality point of view, characterization of the cellular as well as the scaffold component of the graft is of special importance and requires the definition of markers for cell identity, purity, impurities and for cell potency as well as biomechanical testing of structural components like matrices or scaffolds. These pre-clinical studies should be performed in relevant animal models. Since different large animal models bear inherent drawbacks with respect to the prospected outcome and the particular cell-based product, the choice of the animal model should be underlined by a multi-factorial analysis [25]. In terms of marketing approval, the currently best available large animal models for chondrocyte-based products are goat, horse or sheep [26].

The aim of our present study was to evaluate the in vitro differentiation behavior of culture expanded adult ovine chondrocytes in resorbable scaffolds-assisted cartilage grafts. Here we report that the cartilage markers type II collagen and aggrecan are relevant markers for cell identity and potency in the ovine model. Re-differentiation of dedifferentiated ovine chondrocytes in three-dimensional scaffolds-assisted cartilage grafts in vitro as well as the biomechanical characteristics of the scaffold suggests that chondrocytes embedded in polyglycolic acid-fibrin scaffolds are well suited for cell-based, scaffold-assisted cartilage repair.

## Methods

### Isolation and culture of ovine chondrocytes

Articular cartilage from six adult sheep (3–5 years, merino mix) without macroscopic signs of osteoarthritis or other cartilage injuries was used for the study. For cell culture expansion, cartilage was harvested from the femoral and tibial condyle under sterile conditions. Each donor tissue was strictly handled separately. The cartilage was minced and digested for 16h in RPMI 1640 medium (Biochrom) containing 10% fetal bovine serum (FBS, Biochrom), 1.5 U/ml collagenase P (Roche), 500 U/ml collagenase CLS type II (Biochrom), 50 U/ml hyaluronidase (Sigma), 100 μg/ml gentamycin (Biochrom), 100 U/ml penicillin (Biochrom), 100 mg/ml streptomycin (Biochrom), and 250 μg/ml amphotericin B (Sigma). Viable cells as assessed by trypan blue dye exclusion were seeded into cell culture flasks with an initial density of 2x10^5^ cells/cm^2^ in RPMI 1640 supplemented with 10% FBS and penicillin/streptomycin. Medium was changed every 2 to 3 days. After reaching 80% confluence the cells were detached with trypsin/EDTA (Biochrom) and re-plated with a seeding density of 2x10^5^ cells/cm^2^.

### Three dimensional assembly of scaffold-assisted cartilage grafts

After 3 and 5 passages, the cells were detached and re-suspended in Fibrinogen (Tissucol, Baxter) with a cell density of 2.4*10^7^ cells/ml. The cell/fibrinogen suspension was combined with 10 mm x 10 mm x 1.1 mm PGA (polyglycolic acid) scaffolds (provided by BioTissue Technologies GmbH, Freiburg, Germany). After adding a thrombin solution (1:10 v/v PBS; Tissucol; Baxter), polymerisation of fibrinogen was performed at 37°C for 20 min. The grafts (n = 12 per donor, n = 36 in total) were cultured for up to 3 weeks in vitro in RPMI 1640 medium (Biochrom) supplemented with 5% FBS, 100 U/ml penicillin (Biochrom), 100 mg/ml streptomycin (Biochrom) and 50 μg/ml ascorbic acid (Sigma).

### Real-time polymerase chain reaction (PCR)

Tissue and cell cultures derived from three independent donors were homogenized in 1 ml TriReagent (Sigma, Germany). Bromochloropropane was added, incubated at room temperature for 10 minutes and centrifuged at 12.000 g for 15 minutes. The supernatants were used for the isolation of total RNA using the Qiagen Rneasy Mini Kit (Qiagen, Germany) according to the manufacturer’s protocol. RNA was isolated from native cartilage, cultured cells at passage 0, passage 3 and passage 5 as well as after cartilage graft tissue culture for 1 weeks, 2 weeks and 3 weeks (n = 3 each, per point in time). Subsequently, total RNA (3 μg) was reversely transcribed with the iScript cDNA Synthesis Kit according to the manufacturer’s instructions (BioRad). Samples were normalized according to the relative expression level of the housekeeping gene *glyceraldehyde-3-phosphate dehydrogenase* (*GAPDH*). Real-time PCR (i-Cycler PCR System, BioRad) was performed using 1 μl of cDNA sample and the SYBR Green PCR Core Kit (Applied Biosystems) in triplicates. Relative quantitation of marker gene expression (Table 1) was performed and is given as percentage of the GAPDH product.

**Table 1:** Oligonucleotides

**gene name**	**reference sequence**	**oligonucleotides (5‘→3‘) (up/down)**	**product size (base pairs)**
GAPDH	AF02943	TGG AGT CCA CTG GGG TCT TCA CTA/TTG CTG ACA ATC TTG AGG GTA TT	160
aggrecan	AF019758	TCG GGG TAG GTG GCG AGG AA/GGG CGG TTG GGG AGA CTT CAA	150
type ΙΙα1 collagen	AF138883	GCT GGT CAG AGG GGC ATC GTT/AGG GGG ACC TCG GTC TCC AGA T	129
MMP1	AF267156	CAT TCT ACT GAC ATT GGG GCT CTG/GAA TCG TAG TTA TGG CAT CAA AAG T	190
MMP2	NM_001166180	CTT CCA GGG CAC ATC TTA CGA CAG/ACA TGG GGC ACC TTC CGA GTT	160
MMP13	AF267157	TGC ATC CTC AGC AGG TTG AAG C/GTC ATG GGA AGG GTG CTC ATA GG	101
osteocalcin	NM_001040009	AGG GCA GCG AGG TGG TGA AG/GCC GTA GAA GCG CCG ATA GG	173
TIMP1	NM_001009319	ACA GGT CCC AGA ATC GCA GTG AG/CAG CAG CAT AGG TCT TGG TGA ATC C	147
TIMP2	NM_001166186	GGG CCA AAG CGG TCA GTA AGA AG/AGA GGA GGG GGC TGT GTA GAT AAA C	149
TIMP3	NM_001166187	CCA CAC GGA AGC CTC TGA AAG TC/AGG GTA GCC GAA ATT GGA GAG CA	280

*GAPDH* glyceraldehyde-3-phosphate dehydrogenase.

*MMP* matrix metalloproteinase.

*TIMP* tissue inhibitor of metalloproteinases.

### Histology and immuno-histochemistry

For histological and immuno-histochemical analyses, cartilage grafts (n = 3 per donor and per point in time) were embedded in OCT compound, frozen and cryo-sections (10 μm) were prepared. For overall histological evaluation, conventional hematoxylin & eosin staining was used. Proteoglycans were visualized by staining with Alcian Blue 8GS (Roth) at pH 2.5. Nuclei were counterstained with Nuclear Fast Red. Mineralization of the extracellular matrix was visualized by von Kossa staining, followed by counterstaining with eosin. Sections (n = 3) were incubated in the dark at room temperature with 5% silver nitrate for 30 minutes following incubation with 1.7M sodium carbonate/10% (v/v) formalin for 5 minutes. After washing with tap water for 10 minutes, counterstaining was performed with nuclear fast red for 4 minutes. For immuno-histochemical analysis of type II collagens, cryo-sections were incubated for 40 minutes with the primary antibody (rabbit anti-human type II collagen, Acris). Subsequently, sections were processed using the EnVision System Peroxidase Kit (DAKO) according to the manufacturer’s recommendations, followed by counterstaining with hematoxylin (Merck). To verify the presence of type II collagen during expansion culture in monolayer, chondrocytes were seeded on cover slips (Permanox) at a density of 1x 10^4^ cells/cm^2^ directly after cell isolation (passage 0) and at passage 1, 3 and 5. Cells were fixed with acetone/methanol (1:1 v/v) and stained as described above. For analysis of cell viability and homogenous cell distribution in 3D cartilage grafts, propidium iodide/fluorescein diacetate (PI/FDA) staining (Sigma) was used. Grafts (n = 3 per point in time) were rinsed with PBS, incubated for 15 min at 37°C with 2 μg/ml FDA solution, rinsed again with PBS and incubated with 0.1 mg/ml PI solution for 2 min at room temperature. Grafts were analysed using a fluorescence microscope (Olympus AX70).

### Biomechanics

For testing the mechanical strength and degradation of the scaffold, uniaxial tensile tests were performed using a Zwick material testing machine (Zwick, Ulm, Germany). The maximum failure load and tensile strength of the scaffolds (60 mm x 5 mm x 1 mm) was measured under standardized conditions by using a clamp distance of 20 mm and a crosshead speed of 100 mm/min. To characterize the mechanical strength, the ultimate tensile load until failure/rupture of the scaffold was measured. For degradation, the scaffolds were stored in phosphate-buffered saline (Biochrom) at 37°C for up to 10 days. Failure load was measured at day 0 (n = 30, without storage in PBS) and after storage in PBS at 2, 4, 6, 8, and 10 days (n = 30 each).

For measuring the stiffness of scaffolds (diameter 8 mm, thickness 1 mm), compression tests were performed as described previously [27]. In brief, human articular cartilage (n = 2; diameter 18 mm, thickness 2 mm), PGA scaffolds (n = 6) and fibrin-PGA scaffolds (PGA scaffolds immersed with fibrinogen and polymerized by the addition of thrombin, see ‘Three dimensional assembly of scaffold-assisted cartilage grafts‘; n = 6) were tested on a MTS material testing machine. During an applied displacement-time history, the compression of the specimen and the resulting force are measured. The compression tests were carried out in three stages. The test specimens were firstly compressed up to a global strain of 1/3 of the specimen thickness with a displacement velocity of 0.5 mm/s in the loading process, which was followed by the constant compression stage for 20 seconds and the unloading process with the same displacement velocity as in the first stage. All tests were conducted as unconfined compression tests. In order to avoid slipping of specimens perpendicular to the loading axis, rough paper was placed between scaffold and support. The cartilage samples exhibit cragged bone surfaces. For this reason, these specimens were glued on resin to obtain a plane lower surface. Deformations of the resin itself could be neglected in comparison to the compression of the specimens. In order to determine and to compare the structural stiffness of specimens, a global stiffness by means of the stress–strain relation was determined. The global strain was obtained by relation between the specimens’ thickness and its compression. By curve fitting with a linear function of the stress–strain relation during the loading stage in the regime of the compression peak, the inclination of the stress–strain curve, representing a stiffness, could be determined. It should be emphasized that this value is not equal to the Young’s modulus of the material due to the three-dimensional stress state. The determined global stiffness is a measurement for the entire sample structure providing information about its mechanical properties.

### Scanning electron microscopy (SEM)

Scaffolds were fixed in 5% glutardialdehyde for two hours at room temperature. The scaffolds were then dehydrated for 30 minutes each in an ethanol series from 50% up to 100% ethanol. Subsequently, scaffolds were incubated for 12 hours in 100% ethanol. The ethanol was removed in a critical point dryer. For sputter coating, the dried scaffolds were mounted on a stub and coated with gold-palladium. The sputtered scaffolds were evaluated in the scanning electron microscope (Zeiss DSM 950) under a high vacuum mode.

### Statistical analysis

The Kolmogorov-Smirnov method was applied for testing normal distribution of the data. For statistical analysis of the maximum failure load, normal distributed data were further analyzed with the one way repeated measures (RM) analysis of variance (ANOVA) and data not showing a normal distribution with RM ANOVA on ranks. Subsequently, the parametric paired *t*-test or the non-parametric Wilcoxon signed rank test was applied. Differences were considered significant at p < 0.05. For statistical evaluation of differentially expressed genes, the parametric paired *t*-test or the non-parametric Wilcoxon signed rank test was applied. Differences were considered significant at p < 0.05.

## Results

### Ovine articular cartilage and dedifferentiation of ovine chondrocytes in vitro

**Figure 1:**
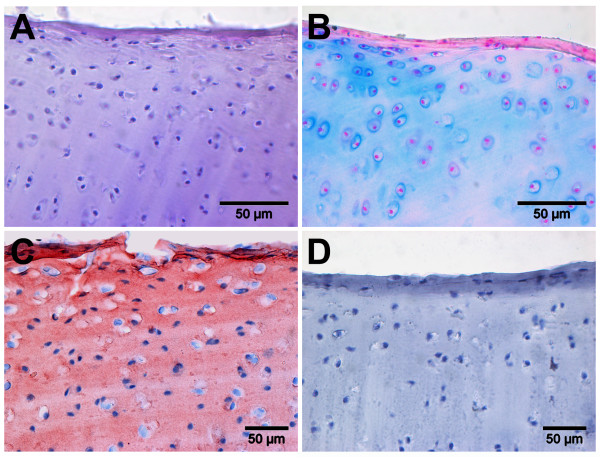
**Histological analysis of typical cartilage extracelluar matrix components in native ovine articular cartilage.** Hematoxylin & eosin (**A**) staining shows that chondrocytes of the articular cartilage are irregularly distributed, with insular cells and cells in groups of two to three chondrocytes. Alcian blue (**B**) staining shows the presence of proteoglycans and an intense staining surrounding the chondrocytes. Type II collagen (**C**) immune-staining is homogenous and shows presence of the cartilage marker molecule in the pericellular extracellular matrix. Controls (**D**), incubated with IgG were negative and show specificity of the type II collagen immune-staining.

Histological staining of ovine articular cartilage (Figure 1) showed irregular distribution of chondrocytes with a characteristic round-shaped morphology. The cells were present as single cells or grow in isogenic groups of two to three cells. The pericellular matrix was smooth and regular (Figure 1A). The chondrocytes were surrounded by cartilage matrix rich in proteoglycan showing a more intense staining adjacent to the lacunae (Figure 1B). Type II collagen that is characteristic for hyaline cartilage is evident and shows a regular and homogeneous distribution within the extracellular matrix (Figure 1C). Controls were negative and show specificity of the type II collagen staining (Figure 1D).

**Figure 2:**
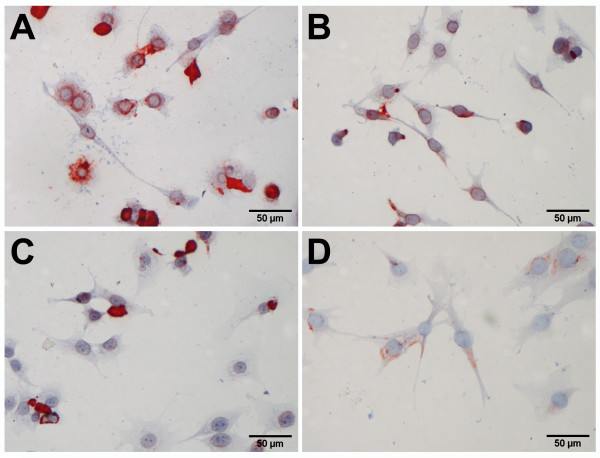
**Immuno-staining of type II collagen in culture expanded, dedifferentiating ovine articular chondrocytes.** Freshly isolated chondrocytes (**A**) in passage 0 attached to the surface of the cell culture flask and show round-shaped as well as already stretch chondrocytes with strong staining of type II collagen. Further cultivation of the chondrocytes up to passage 1 (**B**), passage 3 (**C**) and passage 5 (**D**) revealed stretched cells of a typical fibroblastic appearance and a gradual loss of type II collagen.

For the preparation of cartilage grafts, chondrocytes were harvested by enzymatic digestion of the cartilage matrix and expanded using conventional cell culture conditions (Figure 2). Within a few days, the cells attached to the plastic surface of the cell culture plate. At passage 0, more than 90% of the chondrocytes showed an intense staining of type II collagen (Figure 2A). The cells stretched, developed the shape of elongated, fibrobast-like cells, and progressively lost type II collagen during extensive expansion, at passage 1, 3 and 5 (Figure 2B – 2D).

### Polyglycolic acid-fibrin scaffolds for chondrocyte-based cartilage grafts

**Figure 3:**
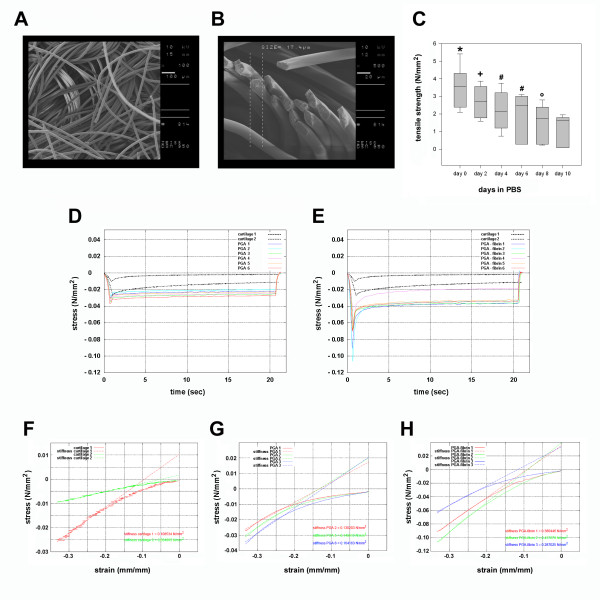
**Electron microscopical and biomechanical characterization of the resorbable, textile polyglycolic acid (PGA) scaffolds.** Scanning electron microscopy shows that non-woven, bulked textile structure of the PGA scaffolds with smooth and regular PGA fibers (**A**). PGA fibers cut with a scalpel show a diameter of approximately 17 μm (**B**). Tensile strength of PGA scaffolds and degradation kinetics in phosphate buffered saline (**C**). Strength is presented as median with the ends of the boxes defining the 25th and 75th percentiles and error bars defining the 10th and 90th percentiles (* p<0.0001 versus day 2–10; + p<0.008 versus day 6–10; # p<0.002 versus day 8–10; **°** p<0.003 versus day 10). Graphical depiction of the measured forces during compression test performed with native articular cartilage as reference tissue and PGA (**D**) as well as PGA-fibrin (**E**) scaffolds. Compression tests and stiffness of human articular cartilage (**F**), PGA scaffolds (**G**) and PGA-fibrin scaffolds (**H**).

Expanded chondrocytes were embedded in fibrin and in resorbable polyglycolic acid scaffolds that ensure initial mechanical stability of the grafts (Figure 3). Scanning electronic microscopy of the PGA scaffold showed a non-woven textile, felt-like structure with regular and smooth PGA fibers (Figure 3A). The fibers were randomly arranged in bundles or singular. The diameter of the fibers was approximately 17μm (Figure 3B). To assess mechanical strength and degradation properties of the scaffolds, the tensile strength were measured before and during the storage of the scaffolds in aqueous solution at body temperature for up to 10 days (Figure 3C). At day 0, the median tensile strength of the PGA scaffold was 3.6 N/mm^2^. The mechanical strength decreased continuously and significantly upon prolonged incubation of the scaffold in an aqueous solution. At day 10, the degrading scaffold showed a median tensile strength of 1.7 N/mm^2^. Compression tests of pure PGA scaffolds (Figure 3D) and PGA-fibrin scaffolds (Figure 3E) started at 0 N/mm^2^ and showed the compression of the specimens up to a global strain of 1/3 of the scaffolds thickness representing the compression peak. At this point, the stress was determined as measured force per loaded area of the specimen. The strength in all scaffolds was higher than in the cartilage sample. PGA-fibrin scaffolds developed the highest stress peaks and consequently the highest strengths. During the constant compression state a relaxation occurred, which was much more pronounced in the case of PGA-fibrin scaffolds than in the tests with pure PGA scaffolds. Consequently, PGA-fibrin scaffolds appeared more capable to dampen stress peaks. Compression tests (Table 2) showed a stiffness of articular cartilage of 0.03 - 0.11 N/mm^2^ (Figure 3F). PGA scaffolds showed a stiffness of 0.13 – 0.16 N/mm^2^ (Figure 3G), while the stiffness of PGA-fibrin scaffolds was 0.29 – 0.44 N/mm^2^ (Figure 3H).

**Table 2:** Stiffness of PGA scaffolds

**No.**	**Sample**	**Stiffness [N/mm**^**2**^**]**
1	human articular cartilage 1	0.1085
2	human articular cartilage 2	0.0341
3	PGA 1	0.1302
4	PGA 2	0.1499
5	PGA 3	0.1642
6	PGA-fibrin 1	0.3805
7	PGA-fibrin 2	0.4377
8	PGA-fibrin 3	0.2870

### Re-differentiation of ovine chondrocytes in polyglycolic acid-fibrin scaffolds

**Figure 4:**
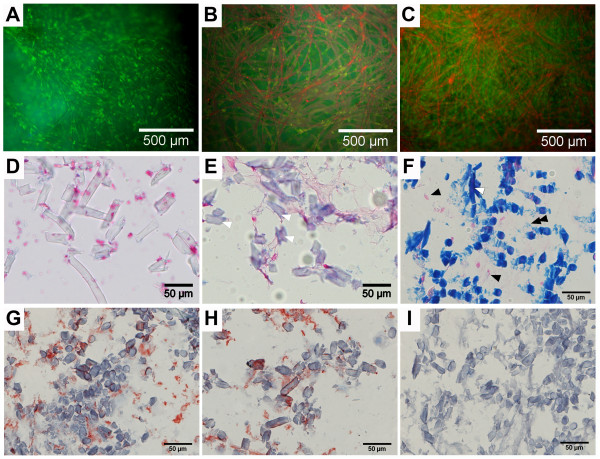
**Histological analysis of ovine chondrocytes in three-dimensional scaffold-assisted cartilage grafts derived from 5**^**th**^**passage chondrocytes.** Propidiumiodide fluoresceindiacetate (PI/FDA) staining shows that the chondrocytes are evenly distributed within the PGA-fibrin scaffold and are vital after 1 (**A**), 2 (**B**) and 3 weeks (**C**) of in vitro cell culture. Alcian blue staining was negative after 1 (**D**) and 2 (**E**) weeks, while the scaffold fibers started to take on the dye after 2 weeks of cell culture (white arrowhead). After 3 weeks (**F**), alcian blue staining shows strong staining of the fragmented PGA fibers (white arrowhead) and vital chondrocytes adjacent to the fibers (black arrowhead). The extracellular matrix shows only marginal staining (black double arrowhead). Immune-staining of type II collagen – formed by chondrocytes expanded up to passage 3 (**G**) and up to passage 5 (**H**) – demonstrated the presence of this particular matrix molecule, typical for articular cartilage. Isotype controls (**I**) gave no signal and show specificity of the type II collagen immune-staining.

To assess the re-differentiation capacity of extensively expanded ovine chondrocytes, cells cultured up to passage 3 or passage 5 (Figure 4) and were embedded in PGA-fibrin scaffolds, grown in three-dimensional tissue culture and evaluated histologically for the presence of cartilage matrix molecules. Although the scaffolds gradually degrade during in vitro tissue culture, the cartilage grafts derived from 5^th^ passage cells were cultured for 3 weeks without losing their shape and still showed viable and evenly distributed chondrocytes (Figure 4A-C). At day 7 (Figure 4A), the scaffold fibers appeared transparent. With gradual degradation of the scaffold, the fibers took on the red color from PI staining at day 14 (Figure 4B) and day 21 (Figure 4C). Histological staining with alcian blue showed that the scaffold fibers remained colorless, not taking on the alcian blue dye at day 7 (Figure 4D). At day 14, the fibers started to fragment and were faintly stained by the alcian blue dye (Figure 4E). After 3 weeks of cell culture, alcian blue staining showed the fragmentation of the PGA scaffolds (Figure 4F, white arrow) and vital chondrocytes growing adjacent to the PGA fibers (Figure 4F, black arrow). The staining of proteoglycan was weak, accumulation of proteoglycan adjacent to or surrounding the cells was not evident (Figure 4F, black double arrow). Re-differentiation of chondrocytes and formation of a cartilaginous matrix in vitro was shown by the staining of cartilage specific type II collagen, formed by chondrocytes expanded up to passage 3 (Figure 4G) and up to passage 5 (Figure 4H). Controls gave no signal and showed the specificity of the antibody staining (Figure 4I). A high viability of chondrocytes in PGA-fibrin scaffolds was evident, when 3^rd^ passage chondrocytes were used (data not shown).

### Gene expression profiles of ovine chondrocytes in vitro

**Figure 5:**
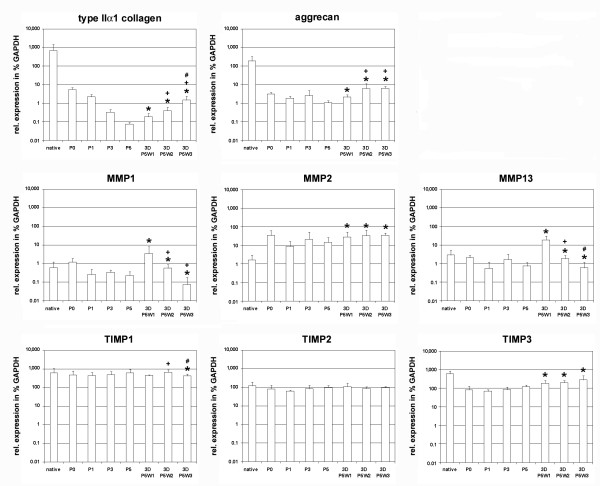
**Semi-quantitative real-time gene expression analysis of ovine chondrocytes in three-dimensional scaffold-assisted cartilage grafts derived from 5**^**th**^**passage chondrocytes.** Chondrocyte dedifferentiation and re-differentiation was analyzed by gene expression analysis of the typical chondrocytic marker genes *aggrecan* and *type IIα1 collagen.* Matrix remodeling was assessed by gene expression analysis of the *matrix metalloproteinases* (*MMP*)*-1*, *-2* and −*13* as well as the *tissue inhibitors of metalloproteinases* (*TIMP*)*-1, -2* and −*3*. The expression level was calculated as the percentage of the expression level of the housekeeping gene *glyceraldehyde-3-phosphate dehydrogenase* (*GAPDH*). The mean (n = 3 donors) is plotted and the error bars represent SD. (*) significant (p<0.05) difference compared to expression level in passage 5; (+) significant (p<0.05) difference compared to expression level in 3D grafts cultured for 1 week; (#) significant (p<0.05) difference compared to expression level in 3D grafts cultured for 2 weeks.

De- and re-differentiation of ovine chondrocytes expanded in vitro were analyzed on the molecular level by real-time gene expression analysis of native cartilage, expanded chondrocytes in passage 0, passage 3, passage 5 and after in vitro tissue culture in PGA-fibrin scaffolds for 1 week, 2 weeks and 3 weeks, using three independent and separately handled donor tissues (Figure 5). Since the re-differentiation capacity of chondrocytes in the cartilage grafts is of special importance for cartilage repair, statistical analysis was performed, comparing the gene expression levels of the respective marker gene in propagated chondrocytes with the levels found in three-dimensional tissue culture in fibrin-PGA scaffolds (Table 3). The gene expression analysis of typical cartilage marker genes *type IIα1 collagen* and *aggrecan* showed a continuous decrease of the expression level during expansion of cells up to passage 5. Re-differentiation of chondrocytes in PGA-fibrin scaffolds was accompanied by a significant (p < 0.05) increase of the cartilage marker gene expression levels compared to the levels found in 5^th^ passage chondrocytes. The expression profiles of matrix modifying molecules like *matrix metalloproteinase* (*MMP*) *-1*, *-2*, and −*13* as well as *tissue inhibitors of metalloproteinases* (*TIMP*) *-1*, *-2*, and −*3* were more heterogeneous. The expression levels of *MMP-1* and *MMP-13* remained stable during the expansion of cells, while the expression level of *MMP-2* increased in cell culture. In the case of *MMP-1* and *MMP-13*, assembly of expanded chondrocytes in PGA-fibrin scaffolds led to a significant increase of the expression levels, with a peak expression after one week in three-dimensional tissue culture. *TIMP-2* showed no differential expression and remained stable during in vitro culture. *TIMP-1* showed a continuous expression level, but a significant decrease after prolonged tissue culture for three weeks compared to the gene expression level found in expanded chondrocytes. The level of *TIMP-3* decreased after the cells were transferred to the monolayer culture (passage 0). Expansion of cells up to passage 5 was accompanied by a continuous increase of *TIMP-3* expression and a significant induction of *TIMP-3* in PGA-fibrin scaffolds compared to the expression level in culture expanded chondrocytes. The gene expression profiles of chondrocytes expanded up to passage 3 and passage 5 showed significant induction of chondrocyte marker genes in three-dimensional tissue culture and revealed initiation of chondrocyte re-differentiation in PGA-fibrin scaffolds (Table 3).

**Table 3:** Statistical analysis of gene expression profiles of 3D cartilage grafts in vitro

**gene**	**point in time**	**rel. expression mean %GAPDH**	**SD**	**p-value vs. 1 week 3D**	**p-value vs. 2 weeks 3D**	**p-value vs. 3 weeks 3D**
**type 2α1 collagen**	P3	0.34	0.15	**0.0066**	**0.0039**	0.0547
	1 week 3D (P3)	1.50	1.00	---	**0.0248**	0.4258
	2 weeks 3D (P3)	3.42	2.23	**0.0248**	---	0.9102
	3 weeks 3D (P3)	5.09	6.78	0.4258	0.9102	---
	P5	0.07	0.06	**0.0104**	**0.0028**	**0.0039**
	1 week 3D (P5)	0.19	0.08	---	**0.0288**	**0.0039**
	2 weeks 3D (P5)	0.40	0.21	**0.0288**	---	**0.0160**
	3 weeks 3D (P5)	1.48	0.84	**0.0039**	**0.0160**	---
**Aggrecan**	P3	2.60	1.91	**0.0153**	**0.0031**	0.0547
	1 week 3D (P3)	6.69	5.47	---	**0.0094**	0.4547
	2 weeks 3D (P3)	16.91	12.05	**0.0094**	---	**0.0039**
	3 weeks 3D (P3)	8.04	8.41	0.4547	**0.0039**	---
	P5	1.05	0.32	**0.0309**	**0.0039**	**0.0002**
	1 week 3D (P5)	2.16	1.11	---	**0.0039**	**0.0039**
	2 weeks 3D (P5)	6.27	4.56	**0.0039**	---	0.9449
	3 weeks 3D (P5)	6.41	2.29	**0.0039**	0.9449	---
**MMP1**	P3	0.34	0.12	**0.0039**	**0.0305**	**0.0008**
	1 week 3D (P3)	3.96	4.48	---	**0.0078**	**0.0039**
	2 weeks 3D (P3)	1.20	1.07	**0.0078**	---	**0.0009**
	3 weeks 3D (P3)	0.13	0.16	**0.0039**	**0.0009**	---
	P5	0.23	0.14	**0.0039**	**0.0029**	**0.0002**
	1 week 3D (P5)	3.52	4.00	---	**0.0039**	**0.0039**
	2 weeks 3D (P5)	0.57	0.30	**0.0039**	---	0.9449
	3 weeks 3D (P5)	0.07	0.10	**0.0039**	0.9449	---
**MMP2**	P3	20.81	26.32	**0.0030**	**0.0032**	**0.0218**
	1 week 3D (P3)	52.38	38.09	---	0.4360	**0.0058**
	2 weeks 3D (P3)	57.71	37.25	0.4360	---	**0.0159**
	3 weeks 3D (P3)	33.18	34.32	**0.0058**	**0.0159**	---
	P5	14.42	10.10	**0.0118**	**0.0232**	**0.0001**
	1 week 3D (P5)	28.02	20.84	---	0.4063	0.2593
	2 weeks 3D (P5)	33.47	28.82	0.4063	---	0.8868
	3 weeks 3D (P5)	34.49	12.90	0.2593	0.8868	---
**MMP13**	P3	1.65	1.42	**0.0078**	**0.0276**	**0.0039**
	1 week 3D (P3)	23.86	18.88	---	**0.0151**	**0.0039**
	2 weeks 3D (P3)	8.68	6.96	**0.0151**	---	0.2867
	3 weeks 3D (P3)	4.78	4.75	**0.0039**	0.2867	---
	P5	0.74	0.38	**0.0008**	**0.0052**	0.0557
	1 week 3D (P5)	19.00	10.39	---	**0.0011**	**0.0008**
	2 weeks 3D (P5)	1.87	1.08	**0.0011**	---	**0.0020**
	3 weeks 3D (P5)	0.60	0.51	**0.0008**	**0.0020**	---
**TIMP1**	P3	497.9	211.8	0.3558	0.7333	**0.0171**
	1 week 3D (P3)	561.2	189.7	---	0.5256	**0.0015**
	2 weeks 3D (P3)	512.1	258.1	0.5256	---	**0.0133**
	3 weeks 3D (P3)	303.6	240.8	**0.0015**	**0.0133**	---
	P5	615.6	334.5	0.2188	0.5559	**0.0391**
	1 week 3D (P5)	463.3	49.7	---	**0.0364**	0.3946
	2 weeks 3D (P5)	676.0	229.4	**0.0364**	---	**0.0023**
	3 weeks 3D (P5)	422.5	121.7	0.3946	**0.0023**	---
**TIMP2**	P3	83.0	46.5	0.2066	0.3109	0.2574
	1 week 3D (P3)	119.4	43.7	---	0.6909	0.0848
	2 weeks 3D (P3)	109.8	48.5	0.6909	---	0.0907
	3 weeks 3D (P3)	63.4	53.1	0.0848	0.0907	---
	P5	97.7	35.1	0.5837	0.4985	0.9227
	1 week 3D (P5)	110.2	55.4	---	0.2950	0.4336
	2 weeks 3D (P5)	86.1	19.3	0.2950	---	0.3490
	3 weeks 3D (P5)	96.2	26.2	0.4336	0.3490	---
**TIMP3**	P3	83.2	30.5	**0.0003**	**0.0008**	**0.0097**
	1 week 3D (P3)	204.0	67.5	---	**0.0061**	**0.0078**
	2 weeks 3D (P3)	156.5	46.4	**0.0061**	---	0.0730
	3 weeks 3D (P3)	99.8	98.9	**0.0078**	0.0730	---
	P5	120.4	35.4	**0.0488**	**0.0195**	**0.0097**
	1 week 3D (P5)	187.2	85.9	---	0.7876	0.1902
	2 weeks 3D (P5)	197.7	69.9	0.7876	---	0.0888
	3 weeks 3D (P5)	282.2	167.3	0.1902	0.0888	---

### Absence of osteogenic differentiation of ovine chondrocytes in polyglycolic acid-fibrin scaffolds

**Figure 6:**
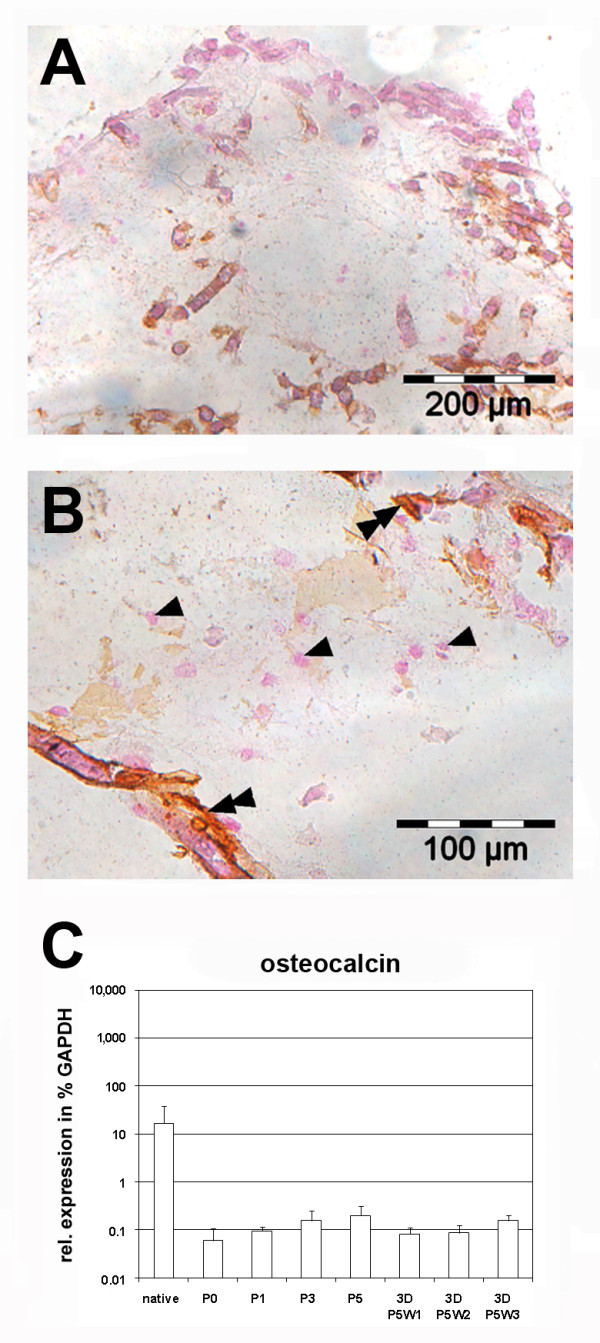
**Histological staining and gene expression analysis of ovine chondrocytes for osteogenic differentiation in three-dimensional scaffold-assisted cartilage grafts.** Von Kossa staining of mineralized osteogenic extracellular matrix was negative (**A**) with mild staining of scaffolds fibers (**B**, black double-arrowhead) and no staining of chondroctyes (**B**, black arrowhead). The gene expression level of the osteogenic marker gene osteocalcin was low (**C**). The expression level was calculated as the percentage of the expression level of the housekeeping gene *glyceraldehyde-3-phosphate dehydrogenase* (*GAPDH*). The mean (n = 3 donors) is plotted and the error bars represent SD.

Since chondrocytes may further differentiate along the osteogenic lineage in PGA-fibrin scaffolds, the presence of osteogenic markers was assessed histologically and by gene expression analysis (Figure 6). Expanded ovine chondrocytes cultured in PGA-fibrin scaffolds for three weeks did not form a mineralized extracellular matrix (Figure 6A). There is a mild staining of the scaffold fibers (Figure 6B, black double arrowheads), while chondrocytes showed no staining (Figure 6B, black arrowhead). In general, the expression level of osteocalcin was low. The mean expression level in native ovine articular cartilage was 16% of the expression level found for the housekeeping gene GAPDH. Expanded chondrocytes showed levels between 0.06% and 1.9%, while chondrocytes embedded in PGA-fibrin scaffolds showed osteocalcin expression levels between 0.8% and 1.6%. There is no evidence that ovine chondrocytes differentiate into bone or osteogenic cells, when expanded or cultured in vitro in PGA-fibrin scaffolds.

## Discussion

In the present study, we used an ovine in vitro model for scaffold-assisted cartilage grafts and showed that extensively expanded chondrocytes lose their chondrocyte phenotype, but re-differentiate in resorbable polyglycolic acid-fibrin polymer scaffolds in vitro as assessed by gene expression analysis and immune-histochemical staining of type II collagen. In addition, our data suggest that the extracellular matrix molecules aggrecan and type II collagen may be suitable markers for chondrocyte identity and potency, regarding the use of the ovine model for pre-clinical animal studies and quality assurance of human cell-based medicinal products for cartilage repair. With respect to secure fixation of scaffold-assisted cartilage grafts in cartilage defects, biomechanical characterization of the scaffolds should comprise testing of tensile strengths.

A variety of studies have documented that chondrocytes dedifferentiate during culture and expansion in monolayer. The cells lose their typical macroscopical and molecular phenotype which is accompanied by the decrease of the cartilage-specific type II collagen and the increase of fibroblast-related type I collagen [28-30]. Three-dimensional re-arrangement of cells initiates cartilaginous re-differentiation of the dedifferentiated chondrocytes, characterized by the re-expression of cartilage marker molecules like aggrecan and type II collagen. However, the in vitro expression levels of chondrocyte marker genes are at least a magnitude lower than found in native articular cartilage. This may indicate that three-dimensional assembly of expanded chondrocytes in PGA-fibrin scaffolds activates the chondrogenic re-differentiation process in vitro, while tissue formation and maturation occurs after transplantation of such grafts in vivo. This capacity of expanded chondrocytes embedded in fibrin and resorbable polymer scaffolds made from PGA or polylactic acid (PLGA) copolymers to form a cartilaginous matrix and cartilage repair tissue in vivo has been shown in various animal studies using nude mice, rabbit and the horse model as well as in clinical trials [22,31-34].

In particular, the markers aggrecan and type II collagen are characteristic for mature cartilage tissue and the dynamics of gene regulation processes during chondrocyte de- and re-differentiation. The synthesis of these markers is suggested to account for phenotypic stability of chondrocytes [35]. Therefore, these molecules are suggested to be suitable markers for chondrocyte identity and potency. The use of type II collagen for testing chondrocyte identity is in accordance with the recommendations of national orthopedic societies for quality assurance in autologous chondrocyte implantation [36]. However, the re-expression of type II collagen by expanded chondrocytes in hydrogel cultures is not predictive for effective formation of cartilage repair tissue in vivo, as assessed in the small animal nude mice model [37].

Interestingly, the re-differentiation of chondrocytes in PGA-fibrin scaffolds was accompanied by induction of MMP-2, TIMP-1 and −3 as well as by a peak increase of MMP-1 and MMP-13 in the early phases of 3D tissue culture that decreased again after prolonged in vitro cultivation. MMP as well as their inhibitors are key enzymes that regulate cell-extracellular matrix interaction during development and differentiation of cartilage. MMP-13 is known to target type II collagen and aggrecan and can be activated by MMP-2. MMP-1 cleaves types I and III collagen and induces MMP-13 in some cell types, including osteoarthritic chondrocytes [38-40]. Particularly in OA, MMP-13 may play an important role in cartilage homeostasis and progression of cartilage breakdown [41,42]. Consequently, an imbalance between matrix degrading enzymes and their tissue inhibitors has been suggested in the development of the disease, as shown by mild cartilage degradation in TIMP-3 deficient mice [43]. MMPs, including MMP-13, as well as further key molecules associated with the onset and progression of OA are expressed in chondrocyte clusters, formed during OA. These clusters with chondrocyte proliferation may implicate a repair response with cell migration and re-population of the diseased cartilage [44]. Since MMP-13 is not present in normal mature articular cartilage but in degenerated chondrocytes [45], the peak expression of MMP in the early phase and the decrease in prolonged tissue culture suggests that three-dimensional assembly of chondrocytes may initiate chondrocyte re-differentiation accompanied by development towards tissue remodeling found in normal articular cartilage. However, further studies have to elucidate the molecular interplay and the mechanisms underlying the balance of formation and tissue remodeling in articular cartilage repair.

From the clinical or practical point of view, cell-based cartilage grafts do not only have to ensure formation of appropriate cartilage repair tissue but should also be easy in handling and allow for secure fixation of the graft in the defect. Therefore, the resorbable scaffold has to be initially stable. As shown here, the PGA scaffold shows a high tensile strength of 3.6 N/mm^2^ and stiffness slightly higher than articular cartilage. The mechanical characteristics of cell-based cartilage grafts cultured in vitro or matured in vivo are approximately an order of magnitude lower than native articular cartilage [46,47]. However, in clinical practice, the primary fixation of the scaffold by gluing, cartilage suture or trans-osseous fixation is of importance. If the fixation of the graft fails, the scaffolds get detached and result in locking or catching of the knee and consequently in re-operations and a poor clinical outcome [12,48]. Since frictional forces are suggested to be the main reason for delamination or loosening of grafts [49], the tensile strength of the scaffolds may be more relevant for clinical applicability than the material’s elasticity or compressive strength. Therefore, measuring of the tensile strength is suggested to be of special importance for quality assurance of scaffold-based cartilage grafts. Polymer-based textile biomaterials like PGA or PLGA scaffolds as well as collagen-based scaffolds show sufficient tensile strength with 10 to 40 N, as shown here and in a previous study [50], which withstand the forces that may occur directly after implantation of the graft and during rehabilitation with typical continuous passive motion (CPM) regime [50,51]. The PGA or PLGA scaffolds ensure initial mechanical stability for secure fixation of the grafts and allow for an initiation of the chondrocytic re-differentiation process in vitro, while scaffold degradation and maturation of mechanical stable cartilaginous tissue occurs after transplantation in vivo [30,34]. This is in accordance with the formation and ‘mechanical’ development of cartilaginous tissue after transplantation of bovine articular chondrocytes embedded in PLGA-fibrin scaffolds, in a subcutaneous mouse model. Stiffness and tensile strength of the newly formed cartilage tissue increased between 6 and 12 weeks after transplantation of the chondrocyte PLGA-fibrin grafts. At 12 weeks, stiffness and strength of the generated tissue was comparable to native septal cartilage and showed 30-50% of the stiffness and strength found in native articular cartilage [46].

## Conclusion

Our data found in the ovine model suggest that chondrocytes embedded in PGA-fibrin scaffolds re-differentiate in in vitro tissue culture without further stimuli like chondrogenic growth factors and that the cartilage markers type II collagen and aggrecan are relevant for testing of cell identity and potency for chondrocyte-based medicinal products. Characterization of the biomechanical properties of the scaffold-assisted cartilage grafts should focus on measuring the tensile strength and, if applicable, the degradation kinetics of resorbable scaffolds.

## Competing interests

ME, KN, UF and CK are employees of TransTissue Technologies GmbH. TransTissue develops products for the regeneration of mesenchymal tissues. All other authors declare that they have no competing interests.

## Authors’ contribution

EM.: Analysis and interpretation, data collection and drafting the manuscript. NK.: Cell differentiation studies and interpretation of data ZB.: Biomechanical testing. FU.: electron microscopy. PD.: ovine chondrocyte cell culture. SM.: Biomechanical testing and interpretation of data, took part in drafting the manuscript. KR.: Interpretation of data, took part in the design of the study and drafting of the manuscript. KC: Design of the study and critical revision of the article. All authors read and approved the final manuscript.
